# 

**DOI:** 10.1192/bjb.2022.69

**Published:** 2023-10

**Authors:** Allan House

**Affiliations:** is Emeritus Professor of Liaison Psychiatry in the School of Medicine, University of Leeds, UK. Email: a.o.house@leeds.ac.uk


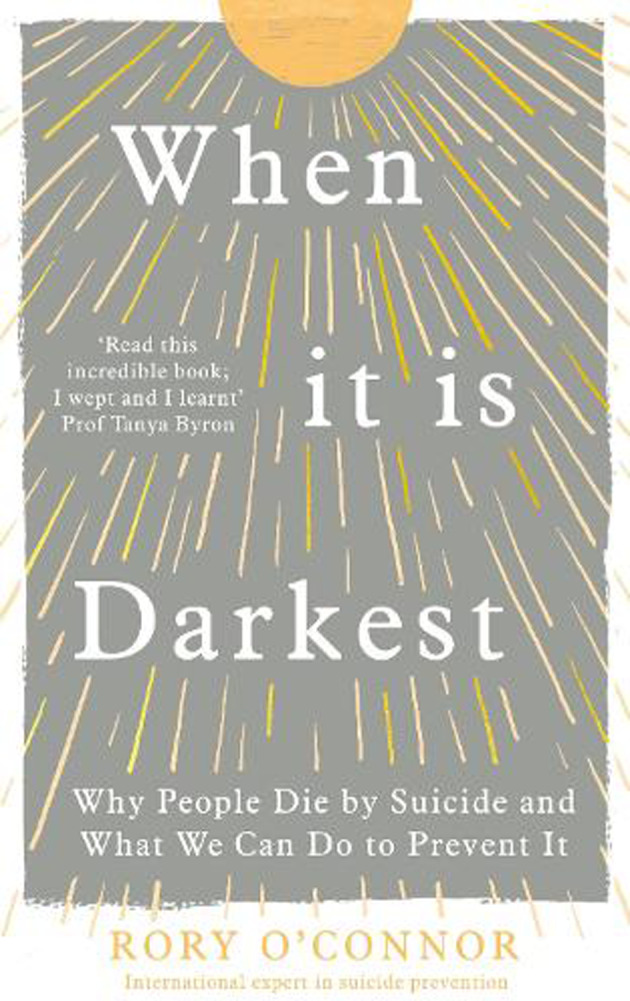


Rory O'Connor is a health psychologist who has published extensively on suicide. He is also active in discussions about suicide aimed at the general public and about suicide prevention policy, especially in Scotland where he lives and works.

His book is divided into four parts, covering the main facts (and misconceptions) about suicide, its main causes, what preventive interventions might be effective and supporting people who are suicidal or who are living in the aftermath of the suicide of somebody close. Further resources are mentioned throughout and there is a list at the end. The emphasis, especially when considering causes, is on the psychology of suicide and includes a review of the author's own framework for organising the disparate associations with suicide into what he calls the integrated motivational–volitional model.

O'Connor's aim is to combine personal and professional perspectives. The style is informal, written in the first person. Interspersed throughout are anecdotes about his personal experiences, his contacts with people who have felt the impact of the suicide of another or of feeling suicidal themselves, and his career in suicide research. At the same time it is in parts quite technical and ends with 48 pages of academic references, with a leaning towards his own research.

The book covers a lot of ground without being exhaustive or exhausting, especially of course in its review of prevailing psychological theories. And it offers a sustained attack against fatalism in the face of suicide and the apparent impossibility of eradicating it: we can move to understand more and to develop effective prevention strategies.

No book like this can be entirely comprehensive but there are some important gaps. There is too little on the personal and social (alienating) impact of drug and alcohol misuse. Mental disorder and its treatment may not be the most important part of suicide prevention but even so it deserves more consideration than it gets. Suicide needs to be seen in social and cultural context if we are to focus public health interventions. In a laudable attempt to combat negativism the effectiveness of suicide prevention interventions is overstated.

What about readership? The book requires quite high levels of general and scientific literacy and that will define its utility. The presentational style will not suit everybody. I personally didn't like the idea of calling suicide The Big S or the proposal that ‘suicide is not usually about the desire to die’, a paradox that will, I fear, be unlikely to appeal to many survivors. Nonetheless this text will find a place as a useful review for the interested non-specialist.

